# Preliminary Study on the Utilization of RHA as a Performance Enhancer for Rubber Mortar

**DOI:** 10.3390/ma14123216

**Published:** 2021-06-10

**Authors:** Jin Li, Peiyuan Chen, Haibing Cai, Ying Xu, Chunchao Li

**Affiliations:** 1 School of Civil Engineering and Architecture, Anhui University of Science and Technology, Huainan 232001, China; 2018100025@aust.edu.cn (J.L.); haibingcai@163.com (H.C.); yxu@aust.edu.cn (Y.X.); 2 Jitoo UHPC(Shandong) New Material Technology Co., Ltd., Shandong Academician Workstation of Ghani Razaqpur, Weihai 264400, China; lcc@mingyangrt.com

**Keywords:** mortar, compressive strength, chloride resistance, capillary absorption

## Abstract

In this study, rice husk ash (RHA) was explored as a strength enhancer for mortars containing waste rubber. The effects of RHA on the flow, mechanical strength, chloride resistance, and capillary absorption of rubber mortar were investigated by substituting up to 20% cement with RHA. The experimental results showed that the incorporation of rubber into mortar could be safely achieved by adding RHA as a cement substitute by up to 20% without compromising the compressive strength of mortar. Moreover, the RHA also exerted positive effects on the enhancement of the chloride resistance as well as the capillary absorption of rubber mortars, for which 15% RHA was found to be the optimal dosage.

## 1. Introduction

The shortage of natural aggregates worldwide has evoked great attention to alternative materials for natural aggregates [1]. This is especially significant in China, where the production of aggregates has sharply decreased in recent years as a consequence of the strict policies of environmental protection [2]. Additionally, recycling wastes into supplements of natural aggregates has boomed in construction sites [3,4]. Currently, recycled aggregates, waste rubber, and plastic are popular recyclable wastes used to replace natural aggregates to prepare concrete [5,6,7]. With proper proportions, concrete incorporated with waste aggregates can display comparable or even superior performance to that with natural aggregates. In particular, waste rubber produced from waste tires [8] has gained attention because of its prominent features, such as its excellent impact resistance, ductility, energy consumption, damping ratio, and toughness [9,10]. Concrete incorporated with waste rubber, namely, rubber concrete, normally has enhanced impact resistance [11], better ductility [12,13], abrasion resistance [14], sound insulation [15], thermal insulation [16,17], and freeze–thaw resistance [18].

However, one of the drawbacks of rubber concrete is the inferior mechanical strength, as rubber is mechanically weaker with a low elastic modulus. In fact, rubber acts as a weak filler in the microstructure of concrete, when stress transfers to the interface between rubber and when the surrounding paste rubber is similar to voids, without the capacity to withstand stress. In addition, the poor interface adhesion between hydrophobic rubber and paste is another reason for the weak mechanical strength of rubber concrete [19,20].

Thus, great efforts have been made toward improving the mechanical strength of rubber concrete [21,22,23]. Positive measurements have been proposed including the use of pozzolanic materials, the introduction of hydrophilic groups on the rubber surface and other pretreatment methods. However, surface modification is complicated and laborsome. In particular, chemical surface modification generates vast amounts of waste, causing pollution to the environment.

Rice husk ash (RHA), a by-product of burned agriculture waste, has been investigated as an effective volcanic ash material [24,25,26]. It is generally believed that when rice husk is burned (<700 °C), a high content of amorphous silica can be produced with a large specific surface area, which can be used as a supplementary cement material (SCM) for concrete applications [24,27]. The influence of RHA on the mechanical properties and durability of mortar has been widely studied. A large number of studies have shown that the addition of RHA into concrete produces three positive effects that optimize the pore structure, secondary pozzolanic reaction, and internal maintenance [25,28,29]. The use of RHA as an active filler by adding it to ultra-high-performance concrete can increase the mechanical properties of concrete, refine the pores, and promote the pozzolanic reaction and the densification of the microstructure [29]. Moreover, the influence of the grain size of RHA on the performance of concrete cannot be ignored. RHA with a fine grain size can help to improve the mechanical properties of concrete and increase its durability. The feasibility of RHA as a supplementary cementitious material in the cement industry has also been evaluated [30].

Currently, it is evident from the work reported above that although a number of studies have examined the properties of plain mortar or concrete with RHA incorporated, the effectiveness of RHA as a performance enhancer on the properties of rubber concrete or mortar has rarely been reported [27,31,32]. For cases in which it is necessary to mitigate the problem of the weak mechanical strength of rubber concrete prior to a practical application, using RHA as a strength enhancer for rubber mortar facilitates the use of RHA as a resource for recycling. Therefore, there is a need to carry out a systematic experimental study to evaluate the effectiveness of RHA as a performance enhancer for the properties of rubber mortars and to provide a reference for the utilization of RHA-reinforced rubber mortar. In this regard, the effects of RHA on the flow, mechanical strength, chloride resistance and capillary absorption of rubber mortars were investigated by substituting up to 20% of cement with RHA (5%, 10%, 15%, and 20%) in this study. The relevant mechanisms were further studied with X-ray diffraction (XRD) and Fourier transform infrared spectroscopy (FT-IR).

## 2. Materials and Methods

### 2.1. Materials

**Figure 1:**
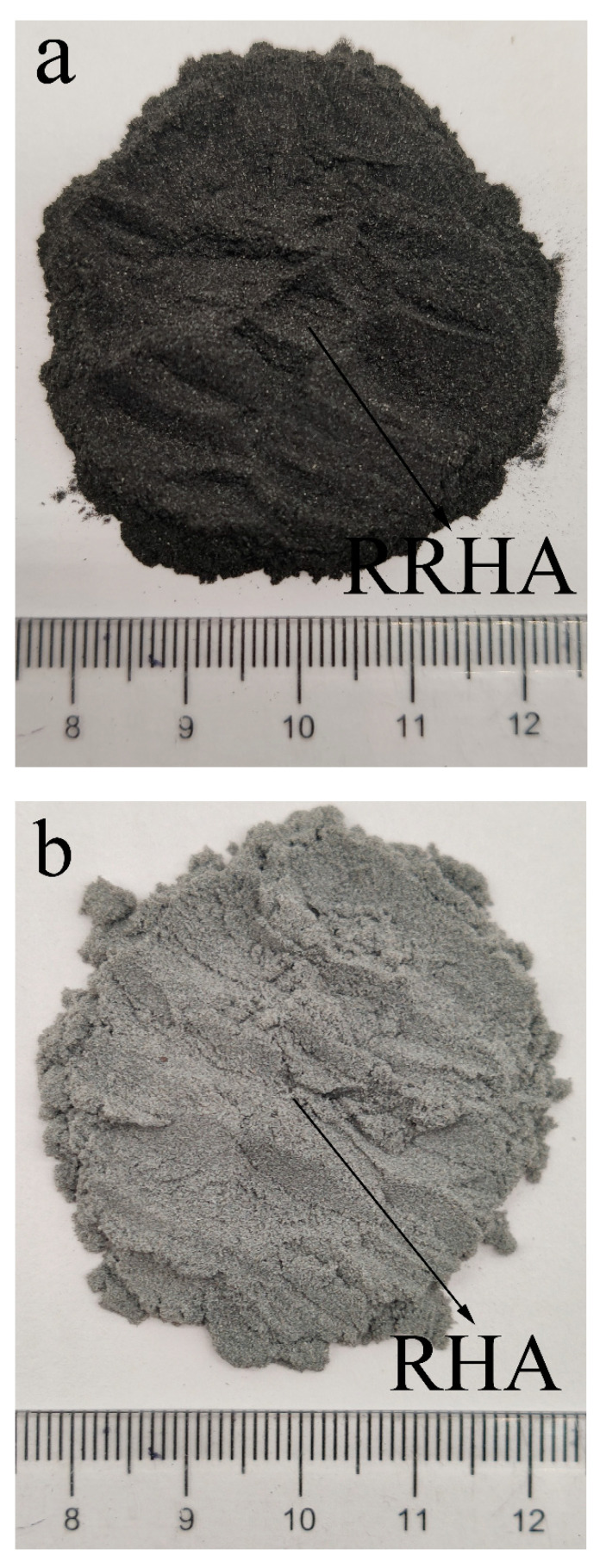
Digital photographs of RRHA and RHA: (**a**) RRHA; (**b**) RHA.

**Figure 2:**
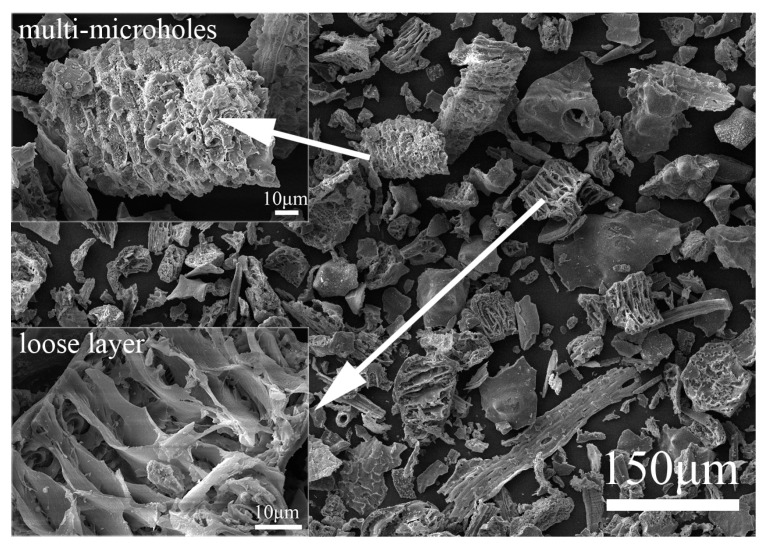
SEM images of RHA calcined at 600 °C for 2 h.

**Figure 3:**
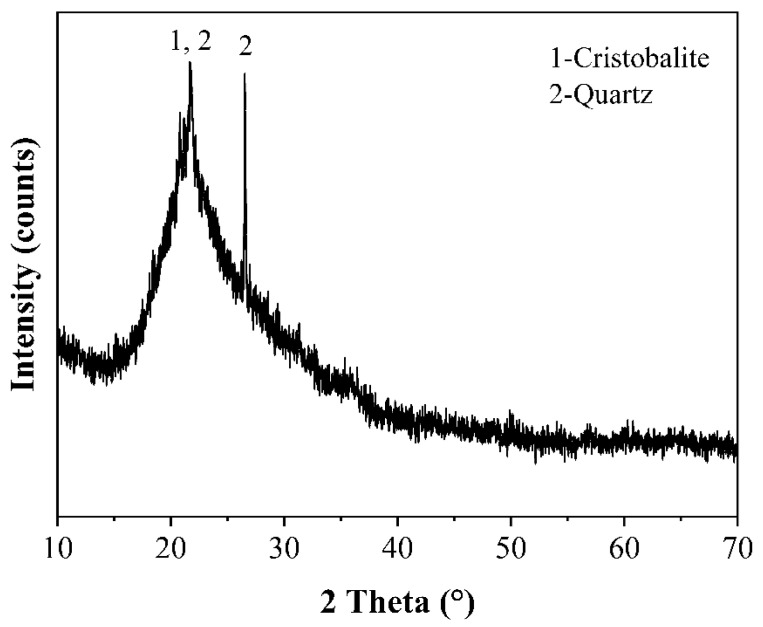
XRD pattern of RHA.

42.5 Ordinary Portland Cement with a 28-day compressive strength over 42.5 Mpa was purchased from Anhui Conch Cement Co., Ltd. Its density and Brunauer, Emmett and Teller (BET) surface area were 3.15 g/cm^3^ and 0.86 m^2^/g, respectively. Raw rice husk ash (RRHA) was provided by the Hubei Xiangyang Grain Factory. The RHA was prepared by calcining the RRHA in a muffle furnace at 600 °C for 2 h to achieve an optimal pozzolanic activity, as proposed by Bie [24]. Table 1 presents the chemical compositions of both the cement and the RHA. It can be seen that the RHA was a classic Si-rich material containing 92% SiO_2_. Figure 1 shows the digital photographs of the RRHA and the RHA. The color change between the RRHA and the RHA indicated the combustion of carbon within the RRHA. Figure 2 further presents the micromorphology of the RHA by scanning electron microscopy (SEM), indicating that the RHA had a very porous structure and loose layers. Consequently, the RHA had a very large BET surface area of 11.071 m^2^/g. Figure 3 presents the XRD pattern of the RHA. Except for small amounts of quartz and cristobalite, the RHA was vitreous in phases, as suggested by the hump peak ranging from 15–35° [33]. To further reveal the content of amorphous silica of the RHA, the Rietveld method was applied using a software program by Jade [34]. The step sizes (0.01°) and the scan speed (1°/min) within 5–80° are typically considered for Rietveld analyses. The conditions used were 40 kV, 50 mA, Cu_Kα radiation, a resolution of 0.0002 and a counting time of 2 s per step. The quantitative analyses of the crystalline and amorphous phases with the Rietveld method were carried out using the Whole Pattern fitting function of Jade. Thus, the amorphous silica content of the RHA was computed with Jade after the fitting process. The results suggested that the crystallinity of the RHA was only 5.59%. In other words, 94.41% of the RHA was in the amorphous phase. To further confirm this finding, the amount of amorphous silica in the RHA was further measured by using a versatile method according to GB/T10846 [35], which provided a standard measuring method for amorphous silica for a variety of supplementary cementitious material, i.e., fly ash, slag and silica fume. Specifically, an XRD analysis of the RHA sample with low scanning speed at 1°/min was first carried out at 15–35°. The XRD pattern was then printed on paper and a baseline was drawn in the peak base. Then, a curve was drawn in the oscillation midpoint of the diffraction intensity curve to separate the vitreous region and the crystal region. The sharp diffraction peak represented the crystal and the rest the vitreous region. Sequentially, the portion of paper with only the profile of the XRD pattern encircled by the baseline printed was cut and weighted, namely, m_1_. Then the portion of paper was further cut to separate the vitreous region, which was also weighted and named m_2_. Finally, Equation (1) could be applied to calculate the content of the amorphous phase of RHA, as follows,

*w_amorphous_*_*phase*_ = m_2_/m_1_ × 100 (1)

where *w_amorphous_*_*phase*_ represents the content of the amorphous phase (%). The result indicated that the *w_amorphous_*_*phase*_ of the RHA was as high as 95%, which agreed well with the Rietveld method.

**Figure 4:**
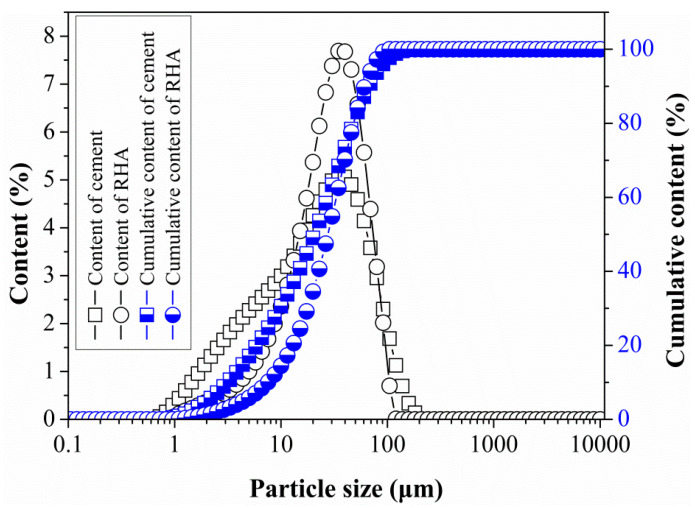
Particle size distribution of cement and RHA.

**Figure 5:**
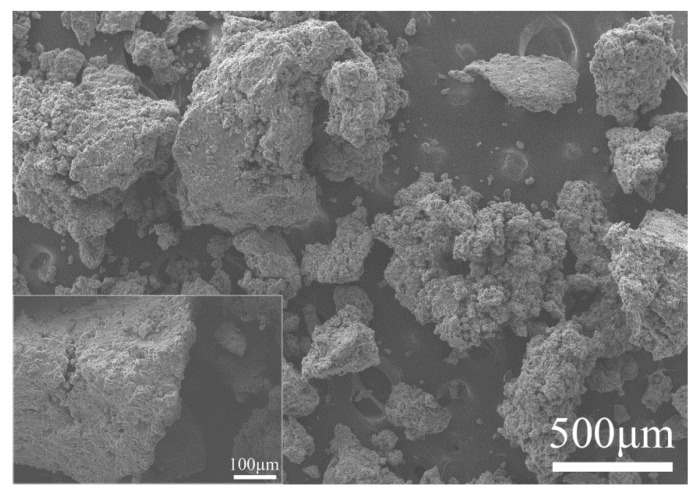
SEM images of rubber.

Figure 4 presents the particle size distribution of the cement and the RHA. 60–80 mesh rubber with particle sizes ranging from 250 μm to 180 μm was used, as shown in Figure 5. The sand was natural river sand with a fineness modulus of 2.36. Tap water was used throughout this research.

### 2.2. Mixing Proportion

Table 2 presents the mixing proportion. Five mixtures were designed with a fixed water-to-cement ratio of 0.5. Additionally, 10% of the sand was replaced with rubber by volume to prepare the rubber mortar. Four replacement levels of RHA to cement by mass were considered at 5%, 10%, 15%, and 20%, respectively. The mortar was prepared following the same procedure. All dry materials were first mixed in a mortar mixer for 5 min. Subsequently, water was added and the mixture was mixed for another 3 min.

### 2.3. Test Methods

#### 2.3.1. Setting Time and Flow

Immediately after mixing, the fresh properties of the pastes prepared according to Table 2 without sand or rubber were tested. Specifically, the Vicat method was adopted to test the setting properties of the pastes according to ASTM C191 [36]. The flow of the mortars was measured according to ASTM C1437 [37] using a flow table.

#### 2.3.2. XRD and FT-IR of Pastes

In order to analyze the microscopic mechanism of the RHA reinforced rubber mortar, the cement pastes cured for 28 days were analyzed with XRD and FT-IR. The mineralogical phase composition of the samples was analyzed with XRD with Cu-Kα radiation whose scanning angle was in the range of 5–60°. FT-IR was used to analyze the molecular vibrations and the chemical bonds in the 28 d pastes. In this study, the FT-IR spectra were obtained from 4000 cm^−1^–400 cm^−1^ since the absorption peaks of interest existed within this wave range. The FT-IR samples were prepared with the KBr tablet method [38].

#### 2.3.3. Capillary Absorption

**Figure 6:**
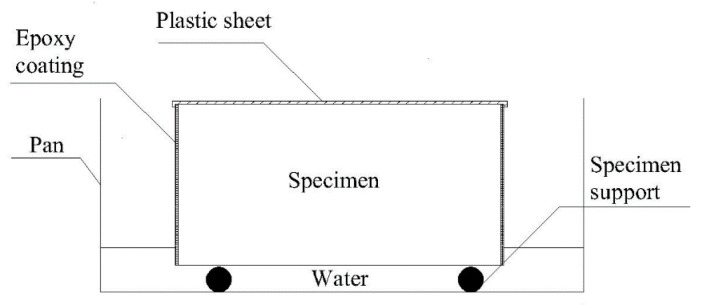
Schematic of the capillary absorption test [40].

The capillary absorption of rubber mortar with and without RHA was tested. According to ASTM C1585 [39], the mixtures were cast into cylinder molds with a size of d × h = 100 mm × 50 mm and cured standardly for 28 d. Prior to testing, the side surfaces of the cylinder specimens were coated with epoxy resin for sealing to ensure one-dimensional water transport. The specimens were then oven-dried to constant weight at 105 °C. Figure 6 illustrates the schematic of the apparatus for the capillary absorption test [40]. During the testing, the upper surfaces of the cylinder sample were sealed with plastic film, and the bottom surface was immersed into water by 1–3 mm.

#### 2.3.4. Compressive Strength and Flexural Strength

According to ASTM C349 [41], the mixtures were cast into plastic molds with a size of 40 mm × 40 mm × 160 mm for measurements of the compressive strength and the flexural strength. The mixtures were initially cured within molds for 24 h under standard curing conditions that at 23 °C and RH > 95%, and subsequently demolded and cured as standard until certain ages. The average results of three specimens were reported for the flexural strength, and the compressive strength was the average value for the six identical tests with the broken specimens.

#### 2.3.5. Chloride Resistance Test

To evaluate the influence of the RHA on the durability of the rubber mortar, chloride resistance was tested according to ASTM C1202 [42]. Fresh mortars were cast into cylinder molds with a size of d × h = 100 mm × 50 mm and cured as standard for 28 days. The side surfaces of the specimens were sealed with epoxy resin. Subsequently, the specimens were vacuum-saturated. The total charge passed for 6 h was tested by a DTL–6 electrical flux tester (Zhongkelujian Co., Ltd., Beijing, China), and the average value of the triplicate samples was reported for each group.

## 3. Results and Discussion

### 3.1. Setting Time and Flow

Table 3 presents the setting times of pastes and the flows of the fresh mortars. The addition of RHA in the mortars caused poor workability as the flow of the mortars decreased according to the dosage of the RHA. In particular, when 20% RHA was added, the flow of R20 was about half of that of R0. The reasons for this variation with the RHA could be attributed to the porous structure and the irregular morphology of the RHA particles [30]. The RHA with a porous structure absorbed the mixing water, and hence increased the consistency of the fresh mortars.

Furthermore, the addition of RHA nevertheless altered the setting times because the more RHA, the shorter the initial and final setting times. The accelerated setting properties of the pastes containing RHA were a result of the reduced effective water–cement ratio due to the highly porous structure of the RHA. This also indicated that partial mixing water was absorbed within the open pores of the RHA. Consequently, the stored water in the RHA could be released as additional feedback to the drying capillary pores to relieve the self-desiccation of cement hydration.

### 3.2. Hydration Products Analyzed by XRD and FTIR

**Figure 7:**
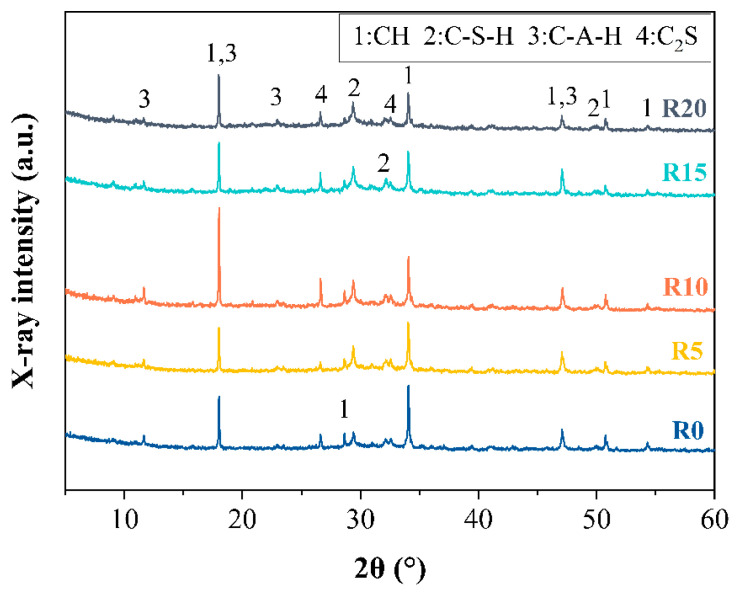
XRD patterns of 28-day pastes.

The hydration products of the 28-d pastes incorporated with RHA were analyzed with XRD, as presented in Figure 7. Four major minerals were detected in all samples, which were portlandite (CH, JCPDS#44-1481), calcium silicate hydrate (C-S-H, JCPDS#33-0306), calcium aluminate hydrate (C-A-H, JCPDS#02-0083), and belite (C_2_S, JCPDS#36-0642).

In general, these XRD patterns were very similar without new minerals being generated when adding RHA to the pastes. The XRD peak heights at 2θ = 34° corresponding to CH were found to decrease with the content of RHA, while the characteristic peak heights of C-S-H at 2θ = 29.4° increased along with the content of RHA [43,44]. Although the quantitative determination of minerals based on XRD peak heights was difficult, especially without an internal reference, these changes in the characteristic peak heights of CH and C-S-H with RHA indicated that the pozzolanic reaction took place between high content of silica contained in the RHA and CH, contributing to the additional generation of C-S-H. This was reasonable and this has been well documented elsewhere [33,45,46,47].

**Figure 8:**
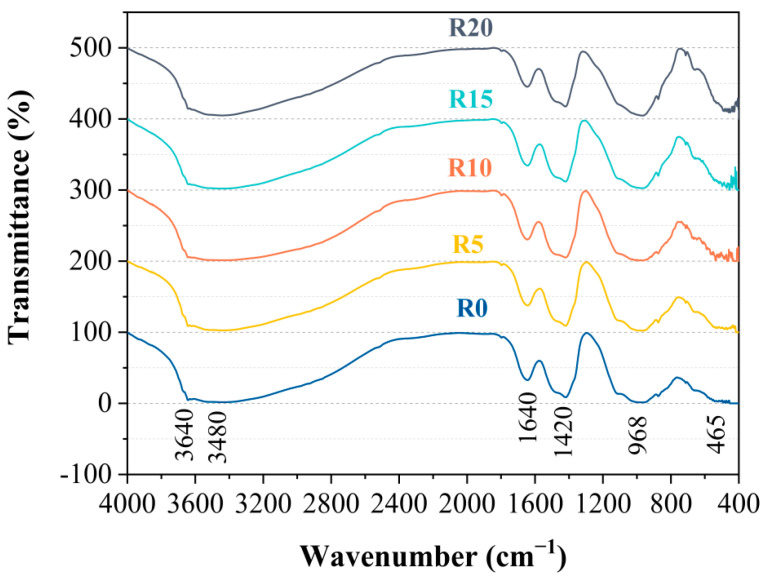
FT-IR spectra of pastes.

Figure 8 shows the FT-IR spectrum of the 28-d pastes. It can be observed from the figure that all of the samples had a wide vibration band near 3480 cm^−1^ due to the stretching vibration of the O–H bond in the adsorbed water [48]. Near 3300 cm^−1^, the internal water of the C-S-H gel generated a vibrating band that overlapped with the vibrating zone caused by the adsorbed water [49]. Furthermore, 3640 cm^−1^ indicated the -OH stretching vibration of the CH [50]. It could be found that the increase of the RHA led to the decreased peak intensity of the CH at 3640 cm^−1^. This was in accordance with the findings by XRD analysis that the pozzolanic reaction of the RHA with CH consumed the content of CH.

There was an obvious acromion vibration band near 968 cm^−1^. According to the study of Yu et al. [48], this was related to the C-S-H gel. The vibration band that occurred near 465 cm^−1^ was caused by the symmetric stretching vibration of the Si–O bond [43]. Similarly, the bending vibration of the O–Si–O bond in the RHA and the vibration of the O–Si–O bond in the C-S-H were also caused by this [43]. The peak that formed at 1640 cm^−1^ was caused by the vibration of the H–O–H bond in the water [51]. Finally, the wavenumber at 1420 cm^−1^ might be attributed to the formation of carbonate during sample preparation [52].

### 3.3. Capillary Absorption

**Figure 9:**
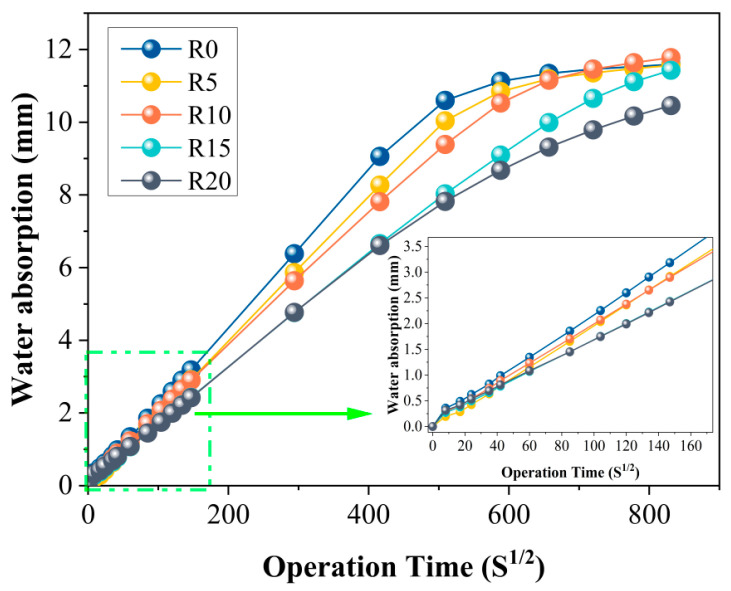
Capillary absorption of mortars.

Figure 9 presents the results of the 9-d capillary absorption experiment. It can be seen from the figure that the addition of RHA reduced the capillary absorption capacity of the rubber mortars despite the fact that the final water absorption of R10 was slightly higher, which might have been a result of the testing error. Moreover, with the increase in RHA, capillary absorption of rubber mortars decreased gradually. This indicated that the microstructure of the rubber mortar was densified by adding the RHA, which was attributed to RHA’s filling effect as well as its pozzolanic nature [53]. In particular, the pozzolanic nature of RHA, mainly composed of amorphous silica, ensured that more hydration products were produced, further filling the pores of mortars and leading to good bonding between the cement matrix and the rubber [54]. In addition, the RHA could be used as a nucleation site in the hydration of rubber mortar, resulting in more C-S-H gel being formed around the RHA. This was reinforced by the XRD results. Therefore, the denser the rubber mortar, the lower the capillary water absorption.

### 3.4. Compressive Strength

**Figure 10:**
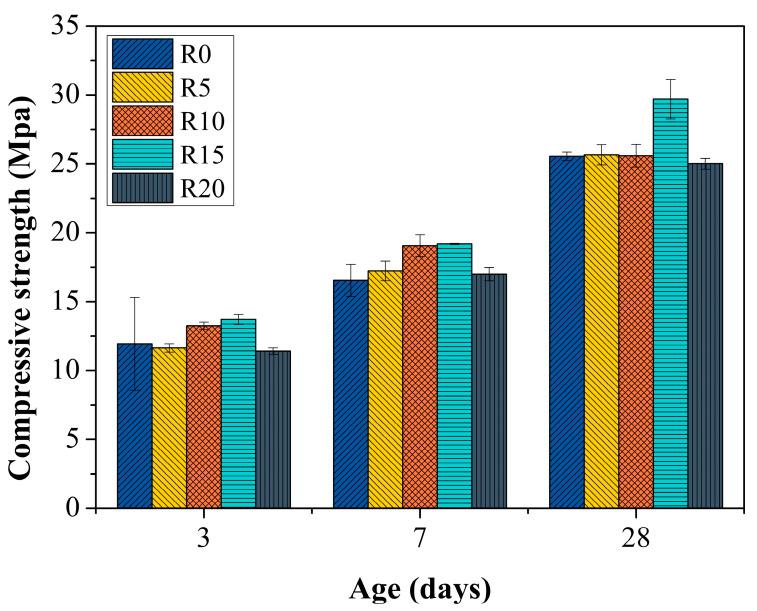
Compressive strength of mortars.

Figure 10 presents the compressive strength of the rubber mortars. When the RHA was added, the compressive strength of the rubber mortar was found to first increase but subsequently decrease along with the dosages of RHA, with R15 as the watershed. Specifically, significant increments of the compressive strength of the RHA-incorporated rubber mortars were gained with 14.82% at 3 d, 16.02% at 7 d and 17.27% at 28 d in R15. The increments of the compressive strength of the RHA-incorporated rubber mortars suggested the advantages of the RHA in compensating for the loss of compressive strength due to rubber. Actually, the RHA functioned as a strength enhancer of the rubber mortar, which could be ascribed to its features of pozzolanic activity and fine sizes. Since amorphous silica is the principal component of RHA, additional hydration products can be produced through the reaction between RHA and CH, resulting in enhanced compressive strength [31]. Furthermore, the fine sizes of RHA are also a reason for the promoted compressive strength [55]. As can be seen from Figure 10, the compressive strengths of R10 and R15 at both 3 d and 7 d were higher than that of R0. In the case of the pozzolanic reaction between the RHA and CH taking a much longer time to exert an obvious influence on the compressive strength of the rubber mortar, this increment of the compressive strength at early ages was thus explained by the fact that RHA, as very fine particles, filled the voids or pores within the microstructure, contributing to a denser matrix and good adherence between the cement matrix and the rubber [33,56].

Nevertheless, 20% RHA seemed excessive in this study because the compressive strengths of R20 were lower than those of R0. This might have been a consequence of the dilutive effect when excessive cement was replaced by RHA [57].

### 3.5. Flexural Strength of Mortar

**Figure 11:**
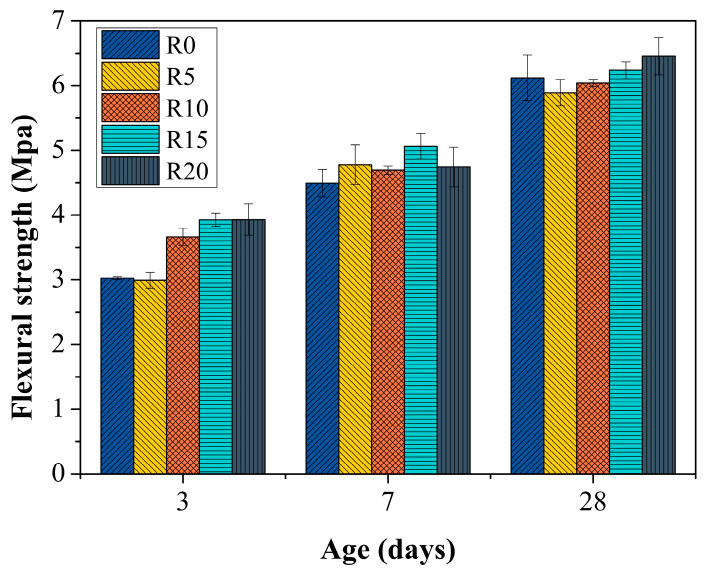
Flexural strength of mortars.

Figure 11 shows the flexural strengths of the mortars. In general, the flexural strength of the RHA rubber mortars was higher than that of R0, especially at 3 d. In fact, more than 21.09–29.92% increments in flexural strength were gained in R10, R15 and R20 compared with that of R0. The greater the RHA content, the higher the flexural strength. This tendency reappeared in the 28-d flexural strength of the RHA rubber mortars despite the fact that the 28-d flexural strengths of R5 and R10 were less than that of R0. The increments of the 28-d flexural strength of R15 and R20 were 1.91% and 5.44%, respectively. At 7 d, although the flexural strengths of all RHA rubber mortars were higher than that of R0, the development tendency with RHA content was different from that of the 3-d and 28-d flexural strengths, but similar to the results for the compressive strength, as presented in Figure 10 [58].

The increase in flexural strength was due to the micro-filling effect and pozzolanic activity [57]. Therefore, the benefit of the RHA for the flexural strength of mortar was thus confirmed [31].

### 3.6. Chloride Resistance

**Figure 12:**
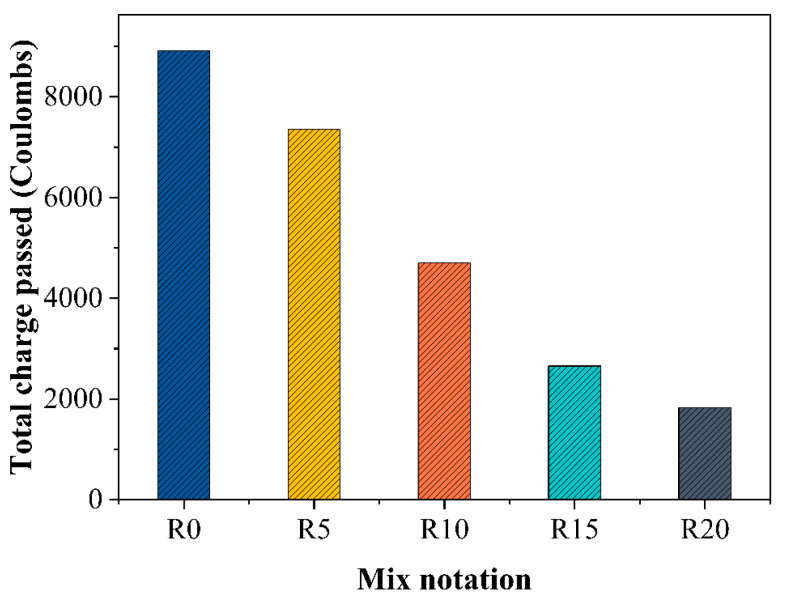
The total charge passed of the cement mortars during 6 h.

Figure 12 presents the results for the chloride resistance of the rubber mortars. The incorporation of RHA exerted an effective influence on the chloride resistance of the rubber mortars, which decreased continuously with the increased content of RHA. Specifically, the total passed charges of R5, R10, R15 and R20 were 17.5%, 47.2%, 70.2% and 79.4% less than that of R0, respectively. This was very consistent with the results of other studies [59,60]. The benefit of RHA for chloride resistance of the mortars was attributed to the pozzolanic activity and the filling effect of the fine RHA particles, as the generated additional hydration products as well as the fine RHA particles contributed to a denser microstructure [61]. Moreover, the pozzolanic reaction between the high content of silica contained in the RHA and the cement generated additional C-S-H gel, resulting in the reduction of the porosity and ultimately the permeability. In such a case, the chloride migration within the microstructure of the rubber mortar could be greatly impeded and the chloride resistance of mortars was enhanced.

## 4. Conclusions

This paper explored rice husk ash (RHA) as a strength enhancer for concrete with waste rubber incorporated. Conclusions were drawn based on the findings, as described below:

(1) The addition of RHA decreased the flowability and accelerated the setting properties of the mortar. An optimal dosage for RHA of 15% was determined based on the flow results.

(2) The benefit of RHA for the compressive strength of mortar was confirmed. It was found that this increased initially, but subsequently decreased with RHA, whereas 15% RHA contributed to the highest compressive strength.

(3) The addition of RHA contributed to a denser microstructure because both the chloride resistance and the water penetration of mortar were greatly decreased. Related mechanisms were summarized as the filling effect and the pozzolanic activity of the RHA, through which the pores of mortar were filled, and additional hydration products could be generated to enhance the compressive strength of mortar.

## Figures and Tables

**Table 1:** Chemical compositions of cement and RHA.

	Chemical Composition/wt.% (XRF)										
	SiO_2_	CaO	Al_2_O_3_	MgO	Fe_2_O_3_	TiO_2_	K_2_O	SO_3_	Na_2_O	P_2_O_5_	MnO
Cement	20.98	61.91	7.69	1.36	3.72	0.44	0.95	2.40	0.20	0.07	0.10
RHA	92.71	0.92	0.48	0.50	0.18	0.04	3.36	0.29	0.08	1.10	0.12

**Table 2:** Mix proportions of mortars (kg/m^3^).

Groups	Cement	Sand	Water	Rubber	RHA
R0	700	945	350	41.23	0
R5	665	945	350	41.23	35
R10	630	945	350	41.23	70
R15	595	945	350	41.23	105
R20	560	945	350	41.23	140

**Table 3:** Fresh properties of pastes and mortars.

Mixtures	Setting Time (min)		Flow (mm)
	Initial Setting	Final Setting	
R0	361	444	266
R5	347	440	238
R10	333	435	214.5
R15	320	428	172
R20	303	418	143

## Data Availability

Data are available on demand by asking the corresponding author.
